# Challenges in Prevention and Care Delivery for Women with Cervical Cancer in Sub-Saharan Africa

**DOI:** 10.3389/fonc.2016.00160

**Published:** 2016-06-28

**Authors:** Thomas C. Randall, Rahel Ghebre

**Affiliations:** ^1^Department of Obstetrics and Gynecology, Harvard Medical School, Boston, MA, USA; ^2^Global Oncology Initiative, Harvard Cancer Center, Boston, MA, USA; ^3^Division of Gynecologic Oncology, University of Minnesota Medical School, Duluth, MN, USA; ^4^Human Resources for Health Program Rwanda, Yale School of Medicine, New Haven, CT, USA

**Keywords:** cervical cancer, sub-Saharan Africa, human resources, surgery, radiation, palliative care

## Abstract

Virtually all cases of invasive cervical cancer are associated with infection by high-risk strains of human papilloma virus. Effective primary and secondary prevention programs, as well as effective treatment for early-stage invasive cancer have dramatically reduced the burden of cervical cancer in high-income countries; 85% of the mortality from cervical cancer now occurs in low- and middle-income countries. This article provides an overview of challenges to cervical cancer care in sub-Saharan Africa (SSA) and identifies areas for programmatic development to meet the global development goal to reduce cancer-related mortality. Advanced stage at presentation and gaps in prevention, screening, diagnostic, and treatment capacities contribute to reduced cervical cancer survival. Cost-effective cervical cancer screening strategies implemented in low resource settings can reduce cervical cancer mortality. Patient- and system-based barriers need to be addressed as part of any cervical cancer control program. Limited human capacity and infrastructure in SSA are major barriers to comprehensive cervical cancer care. Management of early-stage, locally advanced or metastatic cervical cancer involves multispecialty care, including gynecology oncology, medical oncology, radiology, pathology, radiation oncology, and palliative care. Investment in cervical cancer care programs in low- and middle-income countries will need to include effective recruitment programs to engage women in the community to access cancer screening and diagnosis services. Though cervical cancer is a preventable and treatable cancer, the challenges to cervical control in SSA are great and will require a broadly integrated and sustained effort by multiple stakeholders before meaningful progress can be achieved.

## Cervical Cancer and the Way to Meet Global Development Goal

Cervical cancer is a significant cause of cancer-related mortality for women living in sub-Saharan Africa (SSA). In 2013, 39 out of 48 countries, classified as part of SSA region, identified cervical cancer as the most common cause of cancer-related death for women, followed by breast cancer (1). Collectively, the 236,000 women who died from cervical cancer in 2013, 90% of them in developing nations, represent a failure of the health system to implement a functional cervical cancer control strategy (2). The human and societal cost of cervical cancer in SSA is difficult to estimate. The average age at diagnosis is 48; in SSA, most women in this age are subsistence farmers, supporting four to seven or more children. Facilities to treat cervical cancer are scarce in SSA. When surgery and other medical care are available, families face a significant risk of debt and worsened poverty from both the costs of treatment and loss of work (3).

Many challenges stand in the path to develop a health system to address the rising incidence of cancer in SSA, including changing demographics, deficiencies of infrastructure and human capacity, and financial constraints. Multiple steps exist to optimize cervical cancer control: primary prevention with human papilloma virus (HPV) vaccine, secondary prevention with national screening program with HPV DNA test, cytology or visual inspection with acetic acid (VIA), and treatment for invasive cancer. More than 40% of the SSA population is younger than 15 years, and the aging of this population will contribute to a rapidly increasing burden from cervical cancer on these communities (4).

In the context of HPV vaccine where the target age for vaccination of girls and boys is 11 or 12, this young demographic profile in SSA nations can be harnessed to yield greater reward for an investment in primary cervical cancer prevention. The cost of current HPV vaccine is prohibitory for most SSA nations; the GAVI alliance, however, can make this investment more manageable for qualifying nations (5, 6). Models of HPV vaccination in SSA have shown that population-wide programs are highly cost-effective under various circumstances for both the quadrivalent and non-avalent vaccines (7). The current HPV vaccines are heat-labile and, therefore, require an effective “cold-chain” of refrigeration between production and patients. In SSA, where transportation is poor and supply chains can be unreliable, and where 80% of the population still lives in rural settings (World Bank), extensive and coordinated planning is needed to effectively vaccinate a high proportion of the population. The stunning success of the Rwandan vaccination effort is a demonstration of the value of meticulous planning and execution in large-scale implementations (8).

Invasive cervical cancer typically develops 10–30 years after primary HPV infection. Even the most effective vaccination program, therefore, would leave millions of women who are potentially already infected with HPV, at risk for cervical cancer. Screening and cervical cancer treatment are, therefore, critical components of cervical cancer control over the coming generation.

Screening for cervical cancer precursors can be achieved through the use of cytology, HPV DNA testing, or VIA. Cytology-based screening has been the basis of secondary screening in high-income countries for many decades. Cytology-based screening requires an extensive infrastructure, including reliable laboratories with reagents, specialized staff to read the specimens, information systems to notify patients, and caregivers of results and expectations for follow-up, and quality control processes for all of these components. The diagnostic performance of cytology-based testing, furthermore, is highly variable, with limited sensitivity and specificity in even optimal circumstances. The WHO recommends that only countries with established, high-quality programs with broad coverage of their target population utilize cytology-based screening (9).

Visual inspection with acetic acid has been advocated as a low-cost means of population-based screening. The advantages of VIA include limited infrastructure needs, limited initial cost, and an immediate diagnostic result, which in turn allows for “see and treat” programs in which women can be diagnosed and treated for pre-invasive cervical lesions in a single visit, limiting the burden on both patients and the health system by decreasing the need for patient tracking and follow-up (9). VIA has disadvantages as well. It is a subjective test: sensitivity and specificity vary with practitioner performance, making quality control a challenge as programs scale-up from closely monitored research settings to population-based screening. Because of the limited sensitivity of VIA, multiple rounds are needed in a woman’s lifetime. A study led by Shastri and colleagues found that multiple rounds of VIA decreased death from cervical cancer, but did not decrease the incidence of cervical cancer, suggesting that any positive effect may have been more from a stage shift rather than prevention of invasive cancer *per se* (10). In SSA, where resources for the treatment of invasive cancer are limited, this might further decrease the benefit of VIA while increasing the burden on the health-care system and target population. The costs of the health-care work force and facility resources need to be accounted for during any new national cervical cancer screening program. As programs scale-up to population-based screening, robust information systems are needed to manage coverage of those at risk, schedule repeat screening, and avoid redundant testing.

Human papilloma virus testing offers advantages over cytology and VIA. It is an objective test, and, therefore, decreases the demands for human capacity and simplifies quality control. The high sensitivity and negative predictive value of HPV testing makes a single lifetime test a reasonable option for women who test negative.

As articulated by Farmer, global health programs, including in this case effective cervical cancer control, require “space, staff, stuff, and systems” (11). In other words, medical interventions will be sustainably effective and able to respond to crises when a robust health infrastructure is in place. Unfortunately, health infrastructure is a huge challenge in SSA as has been recently elaborated through the work of the Lancet Commission on Global Surgery and others (3, 12). As shown in Table 1, our experience and that of others suggest that the following are salient challenges to cervical cancer control in SSA: clinical diagnostic capacity, capacity for processing and diagnosis of pathology specimens, a lack of oncology specialists at all levels, a deficiency of operating theaters and other surgical services, a lack of radiotherapy equipment and staff (13), and locally contextual factors, including poverty and the financial barriers to treatment, religious and cultural beliefs and stigmas around illness and cancer, and other medical morbidities, particularly coexisting infections, such as HIV, poor nutrition, and obstetrical fistulae. Developing health systems are challenged in resource allocation across many non-communicable diseases. Ultimately, success of any cancer treatment program must take into account the burden of treatment for the patient and her family.

**Table 1:** **Essentials to a cervical cancer management program**.

Elements to consider	
What is the clinical diagnostic capacity?	• Cervical cancer awareness among health professionals • Trained women’s health-care providers (pelvic exam, cervical biopsy)
What is the pathologic diagnostic capacity?	• The who, how, and where of performing cervical disease pathology analysis
Human resources capacity in cervical cancer care	• Trained advanced gynecology surgeons or gynecologic oncologist, radiation oncologist, professional nurses, social workers
Access to cancer surgical services	• Operative room facilities, post-surgical recovery units, access to intensive care units • Surgical support team (nurses, doctors) • Essential surgical supplies and medicines
Radiation oncology facilities	• In-country or out-of-country radiation facility • Limitations due to cost and distance to facility
Contextual modifiers	• Financial barriers for patients and health system • Religious and cultural beliefs toward cancer care • Lack of care givers • Significant medical comorbidities (Urinary obstruction or fistula, HIV status, poor nutritional status)

To date, the majority of cervical cancer control programs in SSA are “vertical” efforts focused on primary or secondary prevention. We use the term “vertical” to describe interventions that are strictly focused on a single disease or condition. Given the paucity of medical infrastructure in SSA, this is most likely the most appropriate choice from the perspective of the single disease. Over time, however, very significant investments have been made in single disease-focused programs without lasting improvement of the overall health infrastructure (14). The U.S. invests $323 million each year for control of HIV in Uganda alone (15), but the sustained effect on medical infrastructure is unclear. Brown and colleagues found that, in Botswana, engagement in HIV treatment services was not associated with a decrease in the typically long interval between initial symptoms and diagnosis of cancer (16). We propose that more effort should be made to strengthen control of diseases within the rubric of overall health system strengthening.

## Treatment for Invasive Cervical Cancer in Sub-Saharan Africa

Primary and secondary prevention of cervical cancer are far more cost-effective than the treatment for invasive cancer and should rightfully be placed as the highest priority of any new cervical cancer control effort. Increasing cancer screening, especially in a context where screening has not been ongoing, will identify women with invasive cancers, incurring an ethical and functional need for management. If a screening program can offer no treatment or comfort for these women, it will both constitute a breach of implied trust and potentially turn the surrounding community against the program and future cancer control initiatives.

The treatment for invasive cervical cancer is determined by the stage of cancer at patient presentation and can range from minor surgery to radical surgery, chemotherapy, and radiotherapy. The development of cervical cancer treatment services requires advanced-level services within a broader integrated health system that includes robust information systems, a functioning consultation and referral network, diagnostic services including pathology and radiology, staffed and functioning operating rooms, perioperative care, radiotherapy services, and chemotherapy. In short, comprehensive management of invasive cervical cancer requires a breadth of services from primary to tertiary care. This raises a conundrum for nations planning a comprehensive cervical cancer control program: the greatest successes will only be seen when a broad and effective health system has been implemented. The exception to this is primary prevention: in our opinion, any effort to control cervical cancer should begin with the steps to implement a comprehensive HPV vaccination program, as this is known to be highly effective. A well-orchestrated campaign in a limited resource health system may succeed before the elements of an integrated and high-quality health system are in place. We do not, however, advocate HPV vaccination as a stand-alone program for cervical cancer control: given the 10–30 interval from HPV infection to the development of invasive cancer, millions of women already infected will be at risk for the next few decades.

The development of screening and treatment for cervical cancer faces significant challenges in SSA, including in information systems, human capacity, and health system infrastructure. Poverty also has a significant effect on cervical cancer control; accommodations should be made to render screening, prevention, and treatment feasible and affordable.

Health information systems in SSA are faced with high rates of illiteracy, limited health-specific knowledge, limited Internet capacity, and limited equipment for medical recordkeeping. Most of the patients in SSA are the keepers of their medical record often traveling with medical cards across clinic. Increasingly, individual hospitals are successfully implementing electronic medical records and thereby facilitate improved coordination of care- and outcome-based research. The use of the OpenMRS platform at AMPATH in Eldoret, Kenya is an excellent example (17). Improved patient identification and health information systems will be needed for cervical cancer control. Recordkeeping is critical to facilitate recruitment and follow-up in screening and prevention and is central to the consultation and referral.

Mobile technology has been found to be effective in improving both recruitment and adherence in the treatment for HIV in SSA (18), and active efforts are underway to refine the role of these technologies in recruitment and follow-up of cervical cancer screening patients. Many countries in SSA have both high rates of cell phone ownership and extensive areas of cellular coverage (19). While mobile technology can be developed to support vertical cervical cancer-specific prevention programs, thought should be given to developing information systems in the context of and in concern with developing electronic medical records in the overall health system.

Treatment for invasive cervical cancer varies with stage at diagnosis. For patients with cancer limited to the cervix, surgery is often the treatment of choice, while for women with more advanced cancer, chemotherapy and radiotherapy are usually needed. Cervical cancer survival is compromised when patients present at advanced stage in high-income countries, where complex multimodality treatments are available. In SSA, where treatment for advanced disease is typically not readily available, this trend is accentuated (12, 20). Delays in presentation and diagnosis can be defined as discrete components: patient delay, health-care providers’ delay, referral delay, and diagnostic waiting time (Table 2) (21). Patient delay in SSA is understandable: due to better treatment for infectious diseases, decreased food insecurity, and an aging population, cancer is a relatively new problem, and many people, especially in remote settings, may have little knowledge of the disease. Even if patients have awareness of cancer, they may risk stigma or financial ruin with diagnosis and treatment. A woman diagnosed with cervical cancer may face abandonment or rejection from her spouse or community (22). Cervical cancer, furthermore, may present with pain bleeding or fistula formation: all issues that may be socially difficult to address in certain contexts. In areas with limited medical care, cancer may often be seen as a “death-sentence,” making diagnosis even less worth the social risk. Even in areas where some level of medical insurance is available, it is common for families to face bankruptcy from medical care (23). Surgery and chemotherapy, when available, often require out-of-pocket expenditures, and time lost from work can be devastating for a patient and her family living on subsistence farming or otherwise in or close to poverty (24). Addressing patient delays, therefore, requires advocacy and public awareness, but more importantly requires structuring the health system and social support such that people are not risking insolvency if they seek care for symptoms or signs of cancer.

**Table 2:** **Delays in diagnosis and treatment for cancer in low resource settings**.

Patient delays	• Limited awareness of cancer • Limited expectation of cure or palliation • Fear of financial ruin • Competing demands for time, money • Distance to treatment facility
Provider delays	• Limited training in cancer diagnosis • Competing/more acute clinical demands • Limited expectation of cure or palliation
Referral Delays	• Unclear referral networks • Absent or unavailable specialists • Limited information systems/medical record technology
Diagnostic delays	• Lack of sufficient pathology facilities and personnel • Backlog of specimens • Limited information systems for results reporting and follow-up with patients and providers

Qualified medical staff are scarce in SSA (25), and those who are available have been trained to manage infectious and other acute illnesses using limited resources; these practitioners often have received limited training in cancer diagnosis and management. There is the potential for patients to present to a medical facility with cancer and to go undiagnosed, leading to a health-care provider delay. In addition, high clinical demands, limited training about cancer and cancer treatment resources, limited information technology, and either an absence or inaccessibility of specialists may lead to referral delays. As discussed earlier, improvements in information technology may help; certainly, there are many examples of the use of connectivity to bring specialty expertise to remote locations (26). In SSA, there are few trained oncology providers; although relationships with outside specialists may help guide care in tertiary centers, it is less clear how to provide guidance for providers at the primary level.

In cases where cancer is suspected, there may be significant diagnostic waiting time. Diagnostic pathology facilities and staff are scarce, and diagnostic testing may be costly for the patient. Often backlogs of specimens develop (27), and this may be exacerbated by limited information systems, making follow-up for results cumbersome.

Late-stage of cervical cancer at presentation in SSA is, therefore, a highly complex multifactorial issue that both arises from and limits the growth of effective cancer control programs. Addressing the various gaps in care will require a comprehensive and integrated approach to each of these factors, from the effects of poverty on family and social structures to the training and availability of oncology specialists.

## Human Resource Capacity for Cervical Cancer Services

Cervical cancer treatment is multimodal. Early stages of cervical cancer can be treated with curative oncologic surgery, whereas advanced or recurrent cervical cancer is best managed with radiation therapy and chemotherapy (28). Current capacity to provide comprehensive women’s cancer care in low- and middle-income countries is constrained by shortage in surgeons trained and experienced in oncologic surgery. The challenge to health-care human resources in LMICs encompasses all levels of the health-care work force, and innovative models to increase the capacity and capability of the health-care work force based on each region-specific conditions are fundamental to any national cancer control program. The current state of the surgical workforce in LMICs is in crisis, directly impacting oncologic surgical services.

The WHO estimates that 57 countries globally face a critical shortage of health professionals, and the number of surgeons and anesthesiologists are particularly scarce (29). Thirty-six of these countries are in SSA, where surgeon density maybe as low as 0.5 per 100,000 people (30, 31). Although the number of health-care work force is only one of the factors impacting surgically treatable conditions, certainly, addressing this dire shortage in surgical work force will advance the neglected area of oncologic global health. An estimated 234.2 million major surgical procedures are performed worldwide each year, 3.5% of the procedures are performed among the poorest one-third of the world’s population, pointing to a large unmet surgical need (32). Limited data exist on the number of oncology specialist in SSA, but reflecting on the state of the health work force in LMIC’s, it can be assumed that health professionals trained in cancer care are soulfully lacking (33). In a report of radiation services in Nigeria, with a population of 160 million and estimated of 100,000 new cancer cases annually, there were 18 radiation oncologist, 8 medical physicists, and 18 radiation therapist to meet the nations radiation therapy (34, 35). Countries such as Rwanda are developing innovative models to meet the challenges, such as task shifting and partnerships, between high resource and low resource cancer centers (36). Beyond the LMIC’s countries, challenges to meet the demands of an aging population on oncology services are anticipated in high-resource countries.

Challenges identified in meeting the demand for essential surgical services in LMIC’s include “brain drain,” the phenomena of losing trained staff from LMICs to high-income countries (37). World Health Organization (WHO) Global Initiative for Emergency and Essential Surgical Care (GIEESC) was launched in 2005 with the goal to scale access and delivery of surgical care in LMICs. The WHO Global Code of Practice on the International Recruitment of Health Personnel recognizes that a strong health system is critical to economic development of a nation and proposed a framework to address the shortage and migration of health-care work force in LMICs (38). A key component of supporting this WHO Global Code is for high-income countries to meet their own demand for health-care force by increasing training in their own country. In addition, a positive outcome of this code is the increased support of high-income countries by providing technical and monetary assistance for addressing health-care force shortage in LMICs.

## Access and Availability of Palliative Services and Medicines in Low Resource Settings

Globally, lack of access to palliative care services and strong pain medications limit the quality of care patients with advanced or end stage cervical cancer can receive. A significant number of women in low resource settings present with advanced cervical cancer. At the time of presentation, cervical cancer symptoms of bleeding, pain, or urinary dysfunction can be debilitating to the patient (39). Approximately 40% of women diagnosed with cervical cancer in a tertiary center in India were staged III and IV (40). In addition, delay in diagnosis and time to initiation of treatment can be significant, resulting in progression of the disease and associated symptoms. Among cervical cancer patients in Ethiopia, 63% of patients were ultimately stage IIB/IV at the time of evaluation for radiation therapy (41). Patients allocated to palliative care group experienced the most significant delay in care (41). A similar pattern of disease presentation has been documented among underserved women in high resource setting (39). The loss of quality of life attributed to cervical cancer diagnosis is not limited to end stage disease. Patients with early-stage cervical cancer undergoing curative intent treatment experience significant anxiety, depression, sexual dysfunction, and treatment side effects, best managed by a multidisciplinary team approach (42).

Palliative care services can be implemented in every resource setting. Team approach to the palliative care is therapeutic to the patient, family, and health-care provider. A nurse, doctor, and social worker are integral to the team. As resources allow, skills provided by physical therapist, pain and palliative care physicians, and oncologist enhance the quality of palliative care services (43). Palliative cancer care provides improvement in quality of life with reduced health-care utilization when implemented early in the course of cancer management (44, 45). Cervical cancer patients may experience loss of appetite, fatigue, vaginal bleeding/hemorrhage, and pelvic pain that improve with initiation of cancer treatment. As cervical cancer progresses, pain, renal dysfunction, and fistulas can be hallmark of the disease. Pain management utilizing the WHO pain ladder remains the standard with incremental increase from non-narcotic to narcotic drugs (46). Establishing access to morphine is critical to alleviating patients suffering. Few global health priorities supersede the critical shortage in pain management in low resource setting (47). A margin 7% of medical use of opioids occurs in middle- and low-income countries, thus compounding barriers to palliative cervical cancer care (48).

## Conclusion

In 2016, the means to prevent and treat cervical cancer are well known and widely available; a death from cervical cancer should be understood as a preventable and unnecessary death. The benefit from vaccination programs will not be realized for decades, leaving millions of women at risk. In areas with limited medical resources, programs of primary and secondary prevention can significantly decrease the burden of cervical cancer. Complete and comprehensive cervical cancer control, however, requires a broadly coordinated effort from multiple specialists and facilities. These specialists can only be trained, and such care can only be safely given, in the setting of a strong overall health system. We propose that outreach efforts in cervical cancer control should broaden their targets beyond process-based and disease-based metrics and work to more broadly strengthen the overall health system.

## Author Contributions

TR and RH wrote this review together in an equal and collaborative fashion.

## Conflict of Interest Statement

The authors declare that the research was conducted in the absence of any commercial or financial relationships that could be construed as a potential conflict of interest.
